# Did a national extended access scheme translate to improvements in patient experience to GP services in England? A retrospective observational study using patient-level data from the English GP patient survey

**DOI:** 10.1186/s12913-025-12447-9

**Published:** 2025-03-08

**Authors:** Liwei Mou, Yiu-Shing Lau, Patrick Burch, William Whittaker

**Affiliations:** 1 Health Organisation, Policy and Economics, Division of Population Health, Health Services Research and Primary Care, School of Health Sciences, Faculty of Biology, Medicine and Health, The University of Manchester, Williamson Building, Oxford Road, Manchester, M13 9PL UK; 2 Public Health Policy and Systems, Institute of Population Health, Faculty of Health & Life Sciences, University of Liverpool, Waterhouse Building, Block B Brownlow Street, Liverpool, L69 3GF UK

**Keywords:** General practice, Patient experience, Service delivery, Access to healthcare, Large dataset, England

## Abstract

**Background:**

Improving access to general practitioner (GP) services is seen as a way to enhance patients’ experiences. England introduced the national extended access scheme to provide routine and urgent GP appointments outside core hours. However, little is known about how this initiative affects patient experience, especially in terms of efficiency and equity. This study aimed to estimate the differential effects of extended access appointments on patient-reported satisfaction, focusing on different service delivery types to assess efficiency and on patient characteristics to evaluate equity of access.

**Methods:**

A retrospective observational study was conducted using data from the English GP Patient Survey (GPPS) (2018 and 2019), linked with data on extended access from NHS records (March 2017 and March 2018). Regression models were used to examine associations between different types of extended access service delivery and patient experience measures (overall experience with GP, satisfaction with appointment time, overall satisfaction with making an appointment, and frequency of seeing or speaking to a preferred GP). Main heterogeneous analyses tested whether effects varied by patient age and employment status. Additional heterogeneous analyses assessed whether the effects differed in patient awareness of services and service providers (GP or GP group where practices collaborate).

**Results:**

The analyses did not identify significant linear associations between extended access services and patient experience measures. However, some evidence suggested that the frequency of seeing or speaking to a preferred GP (a measure of continuity of care) was negatively associated with extended access services, although not linearly. The effect of extended access did not differ by age, but a small positive effect was observed on satisfaction with appointment times for patients in full-time employment. The study also found that greater cooperation between GPs positively impacted patient experience but might compromise continuity of care.

**Conclusions:**

The national extended access scheme had a positive effect on improving satisfaction with appointment times for patients in full-time work, but the effect was not seen across the whole population. The provision of extended access services by GPs at scale may provide additional capacity and choice of care for patients, but care continuity could be threatened.

**Supplementary Information:**

The online version contains supplementary material available at 10.1186/s12913-025-12447-9.

## Introduction

Achieving universal health coverage is one of the key priorities of the Sustainable Development Goals (SDGs) [1, 2]. Good access to health services is a prerequisite for a high-quality healthcare system, especially as a basic function for primary care [3–5]. However, in England, there has been a steady decline of good overall experience of making a GP appointment, suggesting a deterioration in access with subsequent policies aiming to improve access to primary care [6].

Access is a multifaceted concept, comprised of factors related not just to availability of appointments and time of these, but also patient and provider acceptability, affordability, and awareness [7]. Improving access to facilitate universal coverage, therefore, requires a degree of person-centredness [8]. Person-centeredness is recognized as an essential aspect of quality and can be measured through broad indicators, such as patient experience [9]. Patient experience has been found to correlate with clinical quality [10] and healthcare outcomes [11]. Successful improvements in access should, in theory, thus translate into improvements in patient experience.

In England, primary care services are the first point of contact for the healthcare system [12]. In 2023/24, over half (353 million out of 600 million) patient contacts with services commissioned and provided by the NHS were held in primary care [13]. The decline in experience with making GP appointments and the rise in emergency department (A&E) attendance have motivated several schemes designed to incentivize GPs to improve access [14, 15]. In October 2013, the Prime Minister’s Challenge Fund was announced to pilot an ”extended access” scheme for General Practitioners (GP) [15]. The goal of this scheme was to provide additional routine and urgent appointments outside regular GP opening times with a goal of improving access to care to those who were unable to access routine appointments due to work commitments [15, 16].

In April 2016, NHS England set out national plans to ensure that the whole population had access to extended access appointments by 2020, with data on extended access provision collected from October 2016 [17]. The date for full rollout was later brought forward to October 2018. Consequently, data on extended access has not been collected since September 2018 [17]. Clinical Commissioning Groups (CCGs, responsible for commissioning services to a defined local population) commissioned GPs to provide pre-bookable and same-day appointments on weekday evenings (an additional 1.5 h after 6:30 pm), in addition to both Saturdays and Sundays (hours based on local population demand) [6]. NHS England briefing documents shared with CCGs suggested that GPs could collaborate within a hub, and the hubs should focus on providing additional “general practice” capacity. How this was interpreted by different providers varied. However, several features of extended access provision that data suggests were widespread throughout England. As per the model used in the initial pilot of extended access, the ‘hub and spoke’ model of appointment provision was widely used [18]. This was where several nearby practices would offer extended access appointments at one central location [19–22]. The exact type of appointments provided by the hub varied but usually involved face-to-face appointments with GPs or other primary care clinicians. Remote telephone or video appointments were also offered in some extended access settings [23]. In general, patients accessed extended access appointments at a hub in the same manner through which they accessed appointments in their regular practice, usually via the telephone [6]. The scheme, therefore, provided additional capacity and choice of appointments to patients beyond what was previously offered by their general practice. This aligned with a move in NHS England to develop primary care at scale as part of broader integrated services, particularly supporting the integration of extended access to GP services and urgent care services [6]. Similar models, where several GPs coordinate a central hub-based service to provide out-of-hour services have been used in other countries. For example, the GP cooperative model in the Netherlands operates with general practitioners collaborating within a non-profit organization, where they provide out-of-hours care to the shared patient population of all participating doctors [24]. In Slovenia, primary care centres enable patients to seek treatment for minor injuries or illnesses without a need of an appointment [24]. Similarly, in Canada, healthcare reforms in Quebec, Alberta, and Ontario have emphasized group practices and primary healthcare networks to ensure 24/7 access to medical services [25].

The COVID-19 pandemic has exacerbated access problems in GPs in England [26]. Improving patient access to GPs, improving patient experience, and improving access to urgent dental care have been highlighted as one of the four key priorities in the 2025/26 NHS operational plan [27]. Several solutions have been recently proposed to recover accessibility to GPs [28]. For example, patients may also be assigned to within-practice or supra-practice primary care services based on their demographic and clinical characteristics [23, 29]. Additional appointment options such as phone consultations, face-to-face appointments, or online messaging have also been suggested [28]. Investigation of the extended access scheme might provide insights to inform the growing trend towards collaboration across practices.

The extended access appointments scheme has mainly been explored, focusing on the implications for urgent care services, such as using A&Es [30–35]. Whilst several studies have examined the relationship between GP appointment provision outside of core hours and patient satisfaction [14, 36], only one study has evaluated the national extended access policy [22]. They assessed the impact of the national extended access scheme on patient experience by exploiting variations in the number of extended access provision days per week. The paper was, however, limited to practice-level rates of experience, and an inability to assess whether there was variation in the impacts of extended access on different groups of patients. The existing evidence has found significant heterogeneity in age and working status of users of extended access appointments compared to core hour service users [34, 37, 38], implying there may be different impacts on patient experience for these groups. Additionally, this paper did not estimate the heterogeneous effects of different types of service delivery. Some existing evidence has also identified spare capacity in extended access services, particularly on Sundays [34, 37], which suggests that varying types of service delivery could lead to different levels of efficiency. Understanding the heterogeneous effects by patient characteristics and different service delivery models will help inform future delivery models that promote both equity and efficiency in the service.

This study builds on the previous research to provide a more comprehensive evaluation of the impact of extended access appointments on patient experience. It does so by using more granular, patient-level data and broader models of extended access delivery to explore whether there are differential effects on patient experience by different types of service delivery (days, weekdays or weekends of a week), and age and working status. We further conducted a series of additional heterogeneous analyses to assess whether effects differed by patient awareness of services and service provider (GP or GP group where practices collaborate). A range sensitivity analyses were then conducted to assess the robustness of our results.

## Methods

### Data

####  Extended access data

Data on the provision of extended access appointments were sourced from a publicly published dataset by NHS England [39]. The dataset was collected by sending a survey to GP practices in England bi-annually from October 2016 to September 2018, which reflects the cross-sectional information for a one-month collection period. The survey contains questions about the practice and the group of practice if provision is at this level. A group of practices is a collaborative network of practices that provide primary care services to their shared registered population. Such groups have forms like federations or hubs [39]. The dataset information included the types of appointments (early morning or evening appointments on weekdays), the days of provision (Monday to Friday, Saturday, or Sunday), and the way of providing it (through practices or GP groups where practices collaborate) [17]. Information from other datasets was merged to this dataset by NHS England to show the active status of practices and their registered population [40]. To be included as “active practices” in the survey, practices must have active status and more than zero registered populations [40]. Two waves of data (March 2017 and March 2018) were used for the analysis. There were 7428 practices (covering 58.1 million registered patients) in March 2017 and 7276 practices (covering 58.5 million registered patients) in March 2018. 4.3% (320 practices covering 2 million registered patients) in March 2017 and 3.6% (261 practices covering 1.87 million registered patients) in March 2018 did not submit responses.

####  General practice patient survey

Data on patient experience was obtained from the GP Patient Survey (GPPS) with data collection carried out by Ipsos MORI on behalf of NHS England using anonymized patient records [41]. The dataset contains patient-level data, obtained via a data application to NHS England. All data were fully anonymized, with no personal information or identifiable details included. The survey reflects a service evaluation due to its use to evaluate general practice services delivered to registered patients. The provision of the data is contingent on the study using the data for the purposes of which it was collected for, as such, further ethical approval for this study was not required.

GPPS is a routine dataset that records the self-reporting experience of the patients registered with GP services towards primary care. The survey underwent a redesign in 2017 meaning the survey is not comparable prior to this date [41]. Patients eligible for inclusion in the survey met the following criteria: (1) a valid NHS number; (2) continuous registration with a GP practice for at least six months prior to selection; and (3) being 16 years of age or older [41, 42]. The list of practices included patients who met these criteria and whose practices had not opted out of the survey due to concerns about its relevance to their patient population. The sampling process involved two stages [41, 42]. First, NHS Digital provided an anonymised list of patients to determine the sample size and identify individuals for selection [41, 42]. Second, a sample of patients was drawn at the practice level. The survey is sent to eligible patients of a designed sample by post during January and March once per year [41, 42]. The sample frame is designed to achieve an equal distribution of cases across practices through a proportionately stratified (sorted by gender, then age band within each GP practice), uncluttered and random extraction from each GP practice [41]. This means the same person cannot be tracked by different collection periods. After all data are collected, a weighting strategy was applied to the results, aiming at adjusting for differences between all registered patients and the subset of registered patients who completed the questionnaire in the GP practice [42]. GPPS data collected in March 2018 and March 2019 were used for the analysis. The overall response rate was 34.1% (758,165 returned out of 2,221,068 questionnaires sent) in March 2018 and 33.1% (770,512 returned out of 2,328,560 questionnaires sent out) in March 2019 [41, 42].

Responses to the GPPS are likely to present patients’ experiences over the past 12 months; as such, the extended access data collected in March 2017 and March 2018 was used to pair with GPPS results collected in January and March 2018 and January and March 2019 separately. This was chosen to, as best possible, test associations between the effect of provision and patients’ experience over the past 12 months. However, this method requires a strong assumption that the provision of extended access has remained the same between the extended access data collected in March and GPPS data collected during January and March of the next year.

####  Outcome variables: measures of patient experience

As in the study by Burch and Whittaker, patients’ overall experience with GP, and patients’ satisfaction with GP appointment time were used to measure patients’ overall experience and experience of access [22]. Also, in line with the previous study, the ability to contact their preferred doctors was used as a measure for continuity of care [22]. Although continuity has multiple definitions, it is the concept of relational continuity (a patient having a continuous relationship with a clinician) that is measured in the GPPS [43]. We chose to focus on this measure for the following reasons: (1) relational continuity is associated with numerous positive outcomes; (2) patients being seen outside their regular practice (through the ‘extended access hubs’) may reduce relational continuity; and (3) previous studies have examined this measure. Patients’ overall experience with making an appointment was also included as an outcome to measure patients’ experience of access, as the decline of patients’ overall experience with making an appointment is a main drive for the extended access scheme [6]. The outcome measures are shown in Table 1 and the data source is shown in Appendix 3.

**Table 1:** Descriptive of the measures of patient experience included in the analysis

Outcome measures	GPPS questions	Description
Overall experience to GP	Overall, how would you describe your experience of your GP practice?	Patients who answer, ‘Very good’ or ‘Fairly good’ in each practice as binary variable value ‘1’ vs. ‘Neither good nor poor’, ‘Fairly poor’, and ‘Very poor’ as a binary variable value ‘0’
Satisfaction with appointment time	How satisfied are you with the general practice appointment times that are available to you?	Patients who answer, ‘Very good’ or ‘Fairly good’ in each practice as binary variable value ‘1’ vs. ‘Neither good nor poor’, ‘Fairly poor’, and ‘Very poor’ as a binary variable value ‘0’
Overall experience of making an appointment	Overall, how would you describe your experience of making an appointment?	Patients who answer, ‘Very good’ or ‘Fairly good’ in each practice as binary variable value ‘1’ vs. ‘Neither good nor poor’, ‘Fairly poor’, and ‘Very poor’ as a binary variable value ‘0’
Frequencies to see or speak to preferred GP	How often do you see or speak to your preferred GP when you would like to? (This question asked those patients with a preferred GP)	Patients who answer, ‘Always or almost always’ or ‘A lot of the time’ in each practice as binary variable value ‘1’ vs ‘Some of the time’, ‘Never or almost never’, and ‘Have not tried’ as binary variable value ‘0’

####  Control variables: patient and practice characteristics

The models for patient experience adjusted for measures identified in the existing literature to account for observed heterogeneity between patients and practices that may bias the effect on extended access provision. Past literature has identified multiple factors associated with patient experience with GP, including patient characteristics (such as age [44–46], gender [45], ethnicity [44, 45, 47], work status [44], sexual minorities [46, 48], commuting times to work [44], deprivation [44, 46], locations of residence [44, 46], and presence of health conditions [44, 45]), GP practice size [44], distance to practice [49]. The list of control variables and data sources are shown in Appendix 3.

Patient characteristics were mainly sourced from the GPPS data: age, gender, ethnicity, working status, sexual orientation, religion, and long-term physical or mental health conditions. Individuals’ deprivation and rurality levels were sourced from 2019 English indices of deprivation (IMD) and 2011 rural and urban classification [50, 51]. Both datasets were linked to each individual’s small area of residence (LSOA), which, on average, contains about 1,500 people, serving as a proxy for their community. Individuals’ distance to GP was constructed as the Euclidean distance. Northing and easting coordinates for GP clinic’s postcode and population-weighted centroid for each LSOA were used to build Euclidean distance between two points [52, 53].

Additional practice characteristics were sourced from the GP workforce dataset from 2013 to 2019 annual waves to minimise the problem of small samples due to missing data [54]. These included the number of registered patients, the number of full-time equivalent GPs, and the number of full-time equivalent nurses in each GP practice. The workforce dataset for the 2017 annual wave was paired with extended access data collected in March 2017, supplemented by other workforce annual waves. The workforce dataset for the 2018 annual wave was paired with extended access GP data collected in March 2018, supplemented by other workforce annual waves.

### Empirical strategy

####  Sample restriction

The final sample contained patient-level data from 6208 practices, including 316,042 patients from January to March 2018, and 321,741 patients from January to March 2019 (see Appendix 1).

####  Main statistical analyses: by different types of service delivery

Our main analyses estimated the association between the number of extended access days provision and patient experience, using linear probability models of each patient experience measure against extended access measures and patient and practice-level characteristics. The models included two-way fixed effects estimators to control for unobserved practice-specific and time-specific confounders. All models were weighted based on GPPS weights with standard errors clustering at the CCG level. The weighting strategy was applied to make the distribution of individuals representative at the practice level. We used clustered standard errors at the CCG level as practices in each CCG were not independently and identically distributed. STATA (version 16) was applied for analysis.

Further specifications included the number of extended access days on a weekday and a weekend to explore differences in the types of individuals using services via extended access.

####  Main heterogeneous patient group analyses: by patients with age and work status

To examine whether the effects of employment status and age varied, we estimated separate models that included interactions between each patient characteristic and extended access provision. Employment status was included in two ways: as a binary indicator (full-time versus non-full-time) and as a categorical variable (full-time, part-time, or not working). Age was included as a categorical variable.

####  Additional heterogeneous patient group analyses: by patients’ awareness of extended opening times

To address the concern that the respondents of the GPPS may not represent the patients who would have used extended access services at their GP practice, we conducted additional analyses. These analyses included models that examined interactions between patients’ awareness of their practice’s extended opening times (on weekdays or weekends) and the provision of extended access services (on weekdays or weekends).

####  Additional heterogeneous provider analyses: by extended services provided by GP practices or GP groups where practices collaborate

To investigate whether the impact of extended access services varied based on how they were provided (through GP practices or GP groups where practices collaborate), we created a categorical variable with five categories to represent the type of provision during the week, weekdays, and weekends. The five categories are: no provision by either practice or group, provision only by the practice, provision only by the group, provision by both the practice and the group on overlapping days, and provision by both the practice and the group on non-overlapping days.

####  Sensitivity analyses

Four sets of sensitivity analyses were conducted to assess the robustness of the results. First, extended access days were included as a continuous measure (effectively constraining a linear relationship) to test whether the results were sensitive to modelling. The main analysis was re-estimated by modelling extended access days non-parametrically, with separate measures for each volume spanning 1 to 7 days. Second, we used probit regression to account for the binary nature of the outcome variables to assess whether the results were sensitive to the linear probability specification used in the main analyses. Third, the models were estimated using an unbalanced sample, which allowed individuals with only one period of data from the general practice to be included in the sample. Addtionally, we re-estimated the models excluding GP practices with fewer than 1000 registered patients (smaller practices). The final sensitivity analysis incorporated an interactive fixed effect between time and CCGs, which could control for any unobserved shocks caused by other primary care policies.

## Results

### Descriptive analyses

The average number of extended access days provided increased between 2017 and 2018, regardless of different types of service delivery (days (3.4 to 4.3), weekdays (2.6 to 3.3), and weekends (0.8 to 1.1) of a week) (see Table 2). The number and percentage of practices (of total practices) providing full provision of extended days in different types of provision models rose (from 23 to 40%).

**Table 2:** Practices’ provision of extended access services by days in different types of service delivery

The number of extended access days	March 2017, *N* (%)	March 2018, *N* (%)
The number of GP practices	*N* = 6208	*N* = 6208
**Number of extended access days (categories)**		
* 0*	783 (12.6%)	720 (11.6%)
* 1*	1134 (18.3%)	705 (11.4%)
* 2*	974 (15.7%)	617 (9.9%)
* 3*	664 (10.7%)	462 (7.4%)
* 4*	473 (7.6%)	336 (5.4%)
* 5*	446 (7.2%)	394 (6.3%)
* 6*	318 (5.1%)	467 (7.5%)
* 7*	1416 (22.8%)	2507 (40.4%)
* Average* ^a^	3.4 (2.5)	4.3 (2.7)
**Number of extended evening access weekdays (categories)**		
* 0*	1167 (18.8%)	968 (15.6%)
* 1*	1159 (18.7%)	733 (11.8%)
* 2*	977 (15.7%)	636 (10.2%)
* 3*	543 (8.7%)	401 (6.5%)
* 4*	426 (6.9%)	343 (5.5%)
* 5*	1936 (31.2%)	3127 (50.4%)
* Average* ^a^	2.6 (1.9)	3.3 (2.0)
**Number of extended access weekends (categories)**		
* 0*	3062 (49.3%)	2239 (36.1%)
* 1*	1402 (22.6%)	1163 (18.7%)
* 2*	1744 (28.1%)	2806 (45.2%)
* Average* ^a^	0.8 (0.9)	1.1 (0.9)

Different types of service delivery: days, weekdays, and weekends of a week included in the model

^a^Standard deviation in parentheses

Between 2018 and 2019, patients’ satisfaction (‘very satisfied’ or ‘fairly satisfied’) had a slight drop in all outcome measures: overall experience with GP practice decreased from 89.5 to 88.7%, availability of appointment times from 72.8 to 71.3%, experience of making an appointment from 75.6 to 74.1%, and continuity of care (‘always or almost always’ or ‘a lot of the time’) from 55.6–53.2% (see Table 3).

**Table 3:** The number of patients’ responses for each outcome measure included in the model

	January to March 2018, *N* (%)	January to March 2019, *N* (%)
Outcome Measures	*N* = 316,042	*N* = 321,741
**Overall, how would you describe your experience of your GP practice?**		
* ‘Neither good nor poor’*,* ‘Fairly poor’*,* and ‘Very poor’*	33,329 (10.5%)	36,298 (11.3%)
* Very satisfied’ or ‘Fairly satisfied’*	282,713 (89.5%)	285,443 (88.7%)
**How satisfied are you with the general practice appointment times that are available to you?**		
* ‘Neither good nor poor’*,* ‘Fairly poor’*,* and ‘Very poor’*	86,115 (27.2%)	92,250 (28.7%)
* Very satisfied’ or' Fairly satisfied’*	229,927 (72.8%)	229,491 (71.3%)
**Overall**,** how would you describe your experience of making an appointment?**		
* ‘Neither good nor poor’*,* ‘Fairly poor’*,* and ‘Very poor’*	77,108 (24.4%)	83,488 (25.9%)
* Very satisfied’ or' Fairly satisfied’*	238,934 (75.6%)	238,253 (74.1%)
^**a**^ **How often do you see or speak to your preferred GP when you would like to?**		
* ‘Some of the time’*,* ‘Never or almost never’*,* and ‘Have not tried’*	140,291 (44.4%)	150,593 (46.8%)
* ‘Always or almost always’ or ‘A lot of the time’*	175,751 (55.6%)	171,148 (53.2%)

^a^This was asked to patients who have a particular GP they usually prefer to see or speak to

There appeared to be signs of greater collaborations in providing extended access by GP groups where practices collaborate, with increases in the percentage of practices providing extended access services as part of a group. This trend appeared in provision models for days, weekdays, and weekends of a week (see Appendix 2). The average values for explanatory variables in analysis are shown in Appendix 4.

### Main statistical analyses: by different types of service delivery

No association was found between the number of extended access days and any of the four patient experience measures, regardless of any type of service delivery (days, weekdays, and weekends of a week) (see Table 4). Direct distance from home to patients’ registered GP (in kilometres) showed a positive association with all outcome measures across all provision models (for days, weekdays, and weekends), after controlling for extended access days and other covariates. This suggests that distance may be a predictor of patient experience (see Appendix 5, 6, and 7).

**Table 4:** Associations between the number of extended access days and each outcome measure

	Overall experience to GP		Satisfaction with appointment time		Overall experience of making an appointment		Frequencies to see or speak to preferred GP	
	coef.	s.e.	coef.	s.e.	coef.	s.e.	coef.	s.e.
**Number of extended access days**								
* All*	−0.00004	(0.001)	0.0004	(0.001)	−0.0004	(0.001)	−0.0006	(0.001)
* Weekdays*	0.00005	(0.001)	0.0007	(0.001)	−0.0006	(0.001)	−0.0008	(0.001)
* Weekends*	−0.0006	(0.002)	0.00008	(0.002)	−0.0006	(0.002)	−0.0010	(0.002)

*N* = 637783 in each regression. The effect of different types of service delivery (days, weekdays, and weekends of a week) were estimated separately on each outcome measure, using linear probability models. Models controlled for two-way fixed effects, practices, and patients’ characteristics (Full results are included in Appendix 5–7), standard errors are clustered at CCG level

^*^p-value < 0.05 ^**^p-value < 0.01 ^***^p-value < 0.001”, no symbol means *p*-value ≥ 0.05

### Main heterogeneous patient group analyses: by patients with age and work status

The heterogeneous patient group analyses were conducted in a smaller sample, with information available for all control variables. The main model was re-estimated in this smaller sample. The results did not differ significantly from the main regression model (see Appendix 8).

Compared with other work-status patients, full-time work had a negative association with all outcome measures. Patients with part-time work also had a negative association with most outcome measures. The increase of extended access days provision in patients with full-time work had a positive association with patients’ satisfaction with appointment time (0.00184, *p* < 0.05) (see Table 5). This effect strengthened when using a categorical variable (0.00225, *p* < 0.01), where part-time employment status is removed from the base category.

Compared with patients aged 16–24, nearly all age groups had a positive association with all outcome measures with a larger magnitude of coefficient as age increased. No association was found between extended access days provision and any outcome measure in any age group (see Table 5).

**Table 5:** Associations between extended access days and each outcome measure by patients’ work status and age

	**Overall experience to GP**		**Satisfaction with appointment time**		**Overall experience of making an appointment**		**Frequencies to see or speak to preferred GP**		
	**coef.**	**s.e.**	**coef.**	**s.e.**	**coef.**	**s.e.**	**coef.**	**s.e.**	
**Work status as a dummy variable**									
* Number of extended access days*	0.000561	−0.001	−0.000597	−0.001	−0.000969	−0.001	−0.000748		−0.001
* Others (base)*									
* Full-time work*	−0.0223^***^	−0.003	−0.0804^***^	−0.003	−0.0490^***^	−0.004	−0.0310^***^		−0.004
* The interation term between work status (full-time work) and number of extended access days*	−0.000919	−0.001	0.00184^*^	−0.001	0.000886	−0.001	−0.000293		−0.001
**Work status as a category variable**									
* Number of extended access days*	0.000538	−0.001	−0.00101	−0.001	−0.0013	−0.001	−0.000411		−0.001
* Others (base)*									
* Full-time work*	−0.0225^***^	−0.003	−0.0922^***^	−0.004	−0.0562^***^	−0.004	−0.0470^***^		−0.004
* Part-time work*	−0.000585	−0.004	−0.0339^***^	−0.006	−0.0212^***^	−0.005	−0.0405^***^		−0.004
* The interation term between work status (full-time work) and number of extended access days*	−0.000895	−0.001	0.00225^**^	−0.001	0.00122	−0.001	−0.000642		−0.001
*The interation term between work status (part-time work) and number of extended access days*	0.0000995	−0.001	0.00171	−0.001	0.00138	−0.001	−0.00156		−0.001
**Age**									
Number of extended access days	0.0000212	(0.001)	0.000862	(0.002)	0.00116	(0.002)	0.00122		(0.002)
* 16–24 (base)*									
* 25 to 34*	0.0133	(0.008)	0.0285^**^	(0.010)	0.0277^**^	(0.009)	0.0213^*^		(0.009)
* 35 to 44*	0.0441^***^	(0.008)	0.0458^***^	(0.009)	0.0426^***^	(0.008)	0.0242^**^		(0.008)
* 45 to 54*	0.0564^***^	(0.008)	0.0350^***^	(0.009)	0.0231^*^	(0.009)	0.0344^***^		(0.008)
* 55 to 64*	0.0751^***^	(0.007)	0.0641^***^	(0.009)	0.0456^***^	(0.009)	0.0600^***^		(0.008)
* 65 to 74*	0.0940^***^	(0.007)	0.116^***^	(0.009)	0.0856^***^	(0.009)	0.0817^***^		(0.008)
* 75 to 84*	0.117^***^	(0.008)	0.178^***^	(0.010)	0.141^***^	(0.009)	0.0919^***^		(0.008)
* 85 or over*	0.118^***^	(0.009)	0.197^***^	(0.010)	0.147^***^	(0.009)	0.0856^***^		(0.009)
* The interaction term between age (25 to 34) and number of extended access days*	0.0000125	(0.002)	−0.000579	(0.002)	−0.00242	(0.002)	−0.00215		(0.002)
* The interaction term between age (35 to 44) and number of extended access days*	−0.00106	(0.002)	−0.00128	(0.002)	−0.00250	(0.002)	−0.00310		(0.002)
* The interaction term between age (45 to 54) and number of extended access days*	0.000804	(0.001)	0.000242	(0.002)	−0.000325	(0.002)	−0.00201		(0.002)
* The interaction term between age( 55 to 64) and number of extended access days*	0.000479	(0.001)	−0.000949	(0.002)	−0.00236	(0.002)	−0.00285		(0.002)
* The interaction term between age (65 to 74) and number of extended access days*	0.000669	(0.001)	−0.00117	(0.002)	−0.00244	(0.002)	−0.00250		(0.002)
* The interaction term between age ( 75 to 84) and number of extended access days*	0.000234	(0.002)	−0.00181	(0.002)	−0.00292	(0.002)	−0.00100		(0.002)
* The interaction term between age (85 or over) and number of extended access days*	0.000940	(0.002)	−0.00192	(0.002)	−0.000257	(0.002)	−0.000942		(0.002)

*N* = 565018 in each regression for heterogeneous analyses in work status and age groups. The effect of extended access service provision in different work status or age groups was estimated by an interaction term between the number of extended access days in a week and different work status or age groups, using linear probability models. Models controlled for the number of extended access days a week, patients’ work status or age groups, two-way fixed effects, practices, and patients’ characteristics), and standard errors are clustered at the CCG level

^*^p-value < 0.05 ^**^p-value < 0.01 ^***^p-value < 0.001”, no symbol means *p*-value ≥ 0.05

### Additional heterogeneous patient group analyses: by patients’ awareness of extended opening times

Stratified analyses in which patients were aware of their GP’s opening times did not show any association with any of the outcome measures (see Appendix 9).

### Additional heterogeneous provider analyses: by extended services provided by GP practices or GP groups where practices collaborate

The analyses examined whether the type of extended access services provision (through GP practices or GP groups where practices collaborate, by days of the week, weekdays, or weekends) affected outcomes (see Appendix 10). For all analyses, services provided by GP practices were used as the reference category for comparison. Extended access services provided by GP groups on certain weekdays, as well as those offered by both the group and the practice on weekends (when the provision days overlapped), were positively associated with patients’ experiences with their GP. Extended access services provided by both groups and practices on weekends (when the provision days did not overlap) were positively associated with patients’ experiences regarding appointment time and making an appointment. No provision of extended access services on any day of the week was positively associated with the frequency of patients seeing or speaking to their preferred GP.

### Sensitivity analyses

We estimated non-parametric measures of extended access days (see Appendix 11). Compared with no provision of extended access services in the week, any number of days provided per week had a negative association with the measure of patients’ frequencies to see or speak to preferred GPs, although the magnitude of the change in the coefficients was not linear. In addition, our main estimation model was robust to using a probit regression model (see Appendix 12).

We re-estimated the main regression model in two samples: one allowing individuals whose practices have only one period of data, and another with no GP registrations for practices with fewer than 1,000 patients. In both cases, the re-estimates from the main regression model did not differ significantly from the original results. Besides, The estimates were also not significantly different from the main regression results after adding an interaction fixed effect between time and CCGs to the main regression model (see Appendix 13).

## Discussion

### Summary of findings

Using patient-level data, this study aimed to assess whether an extended access to GP services was associated with improvements in patient experience and whether this effect was different for different groups of patients and different methods of service delivery. There was no association found between extended access provision and any outcome measure in any service delivery model (days, weekdays or weekends of a week). Whilst no differential effect was found for patients of different age groups, extended access provision had a small positive effect on patients’ experience with access for those working full-time.

Additional heterogeneous provider analyses found a positive effect on patients’ overall experience with GP practices where there was greater collaboration between practices (GP groups) to provide extended access services during the week and weekends. Similar results were found in patients’ experience with appointment times, where there was greater collaboration between practices to provide extended access services on weekends. However, the provision of extended access by any collaboration between practices had a negative effect on patients’ experience of continuity of care.

Sensitivity analyses found that any provision of extended access days had a negative effect on patients’ experience of continuity of care, although the association was not linear with the increase of provision days.

### Comparison with existing literature

A previous study, using 2018–2019 GPPS practice-level data, examined associations between the number of extended access days provided per week and markers of patient experience with GP, opening time, and continuity of care [22]. There was no association found with any outcome measure in the study at practice-level. This study expanded previous evidence modelling patient-level data and including one extra outcome to measure the patient experience of making appointment. The findings concur with this previous study with no overall association found.

This study further expanded on the work by Burch and Whittaker (2022) by exploring whether there were variations in the effects of patient groups and models of service provision. The findings suggest patients with full-time jobs were more likely to have a better experience with access when the number of extended access days increased. This may be explained by the evidence that patients with full-time work were more likely to use GP out-of-hours services in the context of England, although this evidence was captured based on other schemes to improve GP access [34, 37]. The qualitative results from a case study of two large-scale extended access providers in England had similar results to this study. They found that extended access to GP schemes partly improved the patient experience of GPs with a focus on working people [23]. Our findings may also support the mechanism of the positive effect on the reduction of A&E attendance by extending GP opening times [33, 55].

Our results showed that a greater collaboration in a group of practices had a positive effect on patients experience with GP services and with appointment time, but a negative effect on continuity of care. Any provision of extended access days was negatively associated with continuity of care. These findings are inconsistent with Burch and Whittaker’s [22], who did not find any association. The use of patient-level data, rather than practice-level data, may explain this difference. Our results could be further explained by a qualitative case study, which suggests that greater collaboration between practices (GP groups) may increase access capacity for patients but does not necessarily lead to seamless, integrated care. This is partly because the service is designed for one-off encounters rather than continuous care [23].

### Strengths and limitations

The study is the first to provide more comprehensive evidence in the effects of extended access on patient experience, by using English patient-level data. The study explored the effect by different type of service delivery and tested whether the effect had a differential impact on different groups of the population.

The study has several limitations, which are common to studies using GPPS and extended access data in similar settings [22].

One limitation arises from the design of the GPPS, which means that patients may base their responses on their most recent or accumulated experience of GP services over the course of the year. Although the number of extended days provided by each GP practice or GP group was known, the number of people using the services and the location of a group hub were uncertain. The measures of patient experience relate to experiences with the patient’s practice; the experiences may not therefore reflect experiences of hub engagement. However, we would anticipate the experience of hub engagement to influence a patient’s perception of experience with their practice.

One limitation of this study is the potential correlation between measures of patient experience, as they collectively reflect general levels of satisfaction. This overlap may explain why differences between outcomes across various analyses were relatively small. However, the measures also capture distinct aspects of patient experience, such as overall experience, satisfaction with appointment time, and the experience of making an appointment. The selection of these outcome measures was guided by existing literature, ensuring relevance and validity. Besides, continuity of care contains multiple aspects, but in this study, it is measured using a single question from the GPPS. As a result, the measure of continuity employed here reflects only a limited part of the broader concept of relational continuity, which may include additional dimensions such as sustained interactions over time.

Another limitation lies in the merging of GPPS and extended access data. The extended access data collected in March 2017 and March 2018 was paired with GPPS collected in 2018 and 2019, respectively. The study assumed that there was no change of extended access provision between the collection data of extended access data and followed GPPS completion date. This assumption might undercount the extended days and overestimate the results because most practices are moving towards full coverage.

Finally, the analyses only modelled provision to September 2018 because data was not captured beyond this date.

## Conclusion

The idea of improving out-of-hours GP consultations stem in part from concerns about worsening patient experience in England. These findings need to be considered should extended access (and/or similar collaborative working) schemes be the approach taken to meet the NHS priorities set out in the 2025/26 NHS operational plan [27]. Our findings provided evidence that the national extended access scheme had a positive effect on improving the experience for patients in full-time work, but the effect was not seen across the whole population by any type of service delivery. Given the limited resources available in primary care, the provision of extended access services by GPs at scale might provide additional capacity and choice of care for patients, but relational continuity of care could be threatened in general, which is another key component of quality of care [56] and associated with patient safety [57–59]. The relationship between relational continuity of care and access is not straightforward, but there is evidence to suggest that, once access is taken into account, larger GP practices that collaborate to provide services have lower levels of continuity [60]. Furthermore, among similar models offered in other countries, the GP cooperative model and the primary care centre highlight that the potential advantages are access to services, while the disadvantages are reduced continuity of care [24].

A commonly discussed potential advantage of providing out-of-hours GP services is the reduction in A&E attendance by providing more convenient access for those who are unable to take time off work during core working hours [33, 55]. Several studies have found that the majority of patients attending hubs want to make an appointment in general rather than specifically seeking healthcare outside of normal working hours [20, 21, 23]. This may shed light on why our study did not find any positive impact on patients’ experience of care, and there may be unmet needs that could not be met by improving access via extended GP hours. Those who do not benefit from the extended hours of service may have their continuity of care reduced as a result of the scheme. There is a need for a greater understanding of the unmet needs in GP services delivery.

NHS England has stated that they would invest over £396 million to support the implementation of extended access services across the population [6]. In recent years, there has been a trend towards a shortage of GPs in the workforce, with GP funding under concern and increasing pressure to deliver healthcare services to meet growing demand. GPs are increasingly reluctant to provide healthcare out of hours [61, 62]. The findings here suggest initiatives such as increasing appointment availability into evenings and weekends may only marginally improve access and could raise concerns regarding continuity of care. Policymakers may need to focus on understanding patients’ needs in GP service delivery, as well as considering GPs’ perspectives on providing these services. Policymakers may need to consider additional initiatives to improve access that complement extended access schemes, particularly if improving population-wide access is a priority.

## Supplementary Information

Supplementary Material 1.

## Data Availability

No datasets were generated or analysed during the current study.

## Abbreviations

SDGs: Sustainable Development Goals
A&E: Emergency department
CCGs: Clinical commissioning groups
GP: General Practitioner
GPPS: GP Patient Survey
IMD: Indices of Deprivation
LSOA: Small Area of Residence
