# Supplementary material for: Did a national extended access scheme translate to improvements in patient experience to GP services in England? A retrospective observational study using patient-level data from the English GP patient survey
Source: BMC Health Serv Res. 2025 Mar 8;25:355. doi: 10.1186/s12913-025-12447-9 (PMC11889905; doi:10.1186/s12913-025-12447-9)
Supplement: Supplementary file 1 — Supplementary Material 1. [file 12913_2025_12447_MOESM1_ESM.docx]

**Appendix 1: Data management**

The following process was used to generate the final analysis sample by merging practice-level data with individual-level data.

Two waves of extended access data (March 2017 and March 2018) were used for the analysis.7,428 practices in March 2017 and 7,276 practices in March 2018 submitted data collection responses.4.3% (320 practices) in March 2017 and 3.6% (261 practices) in March 2018 did not submit responses. These practices were still operating at the time closest to data collection and had a higher than zero number of patients registered. We retained only those practices that submitted a response.

GPPS data collected in March 2018 and March 2019 were used in the analysis. The overall response rate in March 2018 was 34.1% (758,165 of 222,068 questionnaires sent were returned) and in March 2019 was 33.1% (770,512 of 233,560 questionnaires sent were returned). We retained individuals who answered all four questions used for the outcome measures in our analyses. In March 2018, a total of 358,896 individuals were registered at 7,242 practices. In March 2019, 355,405 individuals were registered at 6,977 providers. After merging to extended access data, 346,914 individuals were registered at 6,939 practices in the first period and 343,839 individuals were registered at 6,701 practices in the second period.

We then merged individual characteristics including deprivation, rurality and distance of the individual to their GP. The individual’s deprivation and rurality became dummy variables, and their missing data were treated as a category. Missing data for personal distance were excluded as personal distance was treated as a continuous variable. There were 346821 people registered in 6939 practices in the first period and 343706 people registered in 6701 practices in the second period.

In addition, we merged labour force characteristics. We first merged the labour force dataset from the 2017 annual wave with period 1, and the labour force dataset from the 2018 annual wave with period 2. However, there was much missing data. We supplemented the main merged data with other annual waves of the workforce from 2013 to 2019 to minimise small sample issues due to missing data. There were 321,351 people registered at 6,330 practices in period 1 and 327,367 people registered at 6,330 practices in period 2.

We were concerned about individuals whose CCGs were reconfigured between March 2017 and March 2018, as there could be potential confounding factors at these practices. We have therefore excluded individuals who were registered with these practices in both periods. In addition, there were a number of individuals whose registered practices had data for only one of these periods. These individuals were also excluded because of potential confounding factors.

The final sample contained patient-level data from 6208 practices, 316042 patients in period 2, and 321741 in period 2.

**Appendix 2: The way of extended access services provided by practices and groups in different types of service delivery (days, weekdays, and weekends of a week) in the sample**

| **How practices provided extended access service** | **First Period, N (%)** | **Second Period, N (%)** |
| --- | --- | --- |
| **The number of GP practices** | **N=6208** | **N=6208** |
| **How practices provided extended access services: for a total week** |  |  |
| *No extended days provided* | 783 (12.6%) | 720 (11.6%) |
| *Only practice provided extended services for certain number of days* | 3306 (53.3%) | 2073 (33.4%) |
| *Only group provided extended services for certain number of days* | 387 (6.2%) | 755 (12.2%) |
| *Group and Practice overlap in providing extended services* | 1480 (23.8%) | 2479 (39.9%) |
| *Group and Practice did not overlap in providing extended services* | 252 (4.1%) | 181 (2.9%) |
| ***How practices provided extended access services: for weekdays*** |  |  |
| *No extended days provided* | 1167 (18.8%) | 968 (15.6%) |
| *Only practice provided extended services for certain number of days* | 3310 (53.3%) | 2123 (34.2%) |
| *Only group provided extended services for certain number of days* | 441 (7.1%) | 893 (14.4%) |
| *Group and Practice overlap in providing extended services* | 1266 (20.4%) | 2191 (35.3%) |
| *Group and Practice did not overlap in providing extended services* | 24 (0.4%) | 33 (0.5%) |
| ***How practices provided extended access services: for weekends*** |  |  |
| *No extended days provided* | 3062 (49.3%) | 2239 (36.1%) |
| *Only practice provided extended services for certain number of days* | 1114 (17.9%) | 714 (11.5%) |
| *Only group provided extended services for certain number of days* | 1455 (23.4%) | 2323 (37.4%) |
| *Group and Practice overlap in providing extended services* | 575 (9.3%) | 931 (15.0%) |
| *Group and Practice did not overlap in providing extended services* | 2 (0.0%) | 1 (0.0%) |

**Appendix 3: Data source for variables used in the analyses**

|  | **Data source** | **Data level** |
| --- | --- | --- |
| **Outcome measures** | | |
| Overall experience to GP | GP Patient Survey dataset collected in March 2018 and March 2019 | Patient level |
| Satisfaction with appointment time |  |  |
| Overall experience of making an appointment |  |  |
| Frequencies to see or speak to preferred GP |  |  |
| **Explanatory variable** | | |
| The number of extended access days | Extended access to GP dataset collected in March 2017 and March 2018 | Practice level |
| **Covariates** | | |
| Age | GP Patient Survey dataset collected in March 2018 and March 2019 | Patient level |
| Gender |  |  |
| Ethnicity |  |  |
| Working Status |  |  |
| Sexual Orientation |  |  |
| Religion |  |  |
| Long-term health conditions |  |  |
| Indices of Deprivation | 2019 English indices of deprivation (IMD) | Patient level |
| Rurality | 2011 rural and urban classification | Patient level |
| Total patients | The GP workforce dataset collected in March 2017 and March 2018, supplemented by other annual wave from 2013-2018 | Practice level |
| Total number of FTE GPs per 10,000 patients |  |  |
| Total number of FTE Nurses per 10,000 patients |  |  |
| Direct distance from home to their registered GP (km) | GP Patient Survey dataset collected in March 2018 and March 2019 | Patient level |

**Appendix 4: Descriptive statistics on covariates in the model**

|  | **First Period, N (%)** | **Second Period, N (%)** |
| --- | --- | --- |
| **Age** |  |  |
| *16-24* | 13433 (4.3%) | 13296 (4.1%) |
| *25 to 34* | 21956 (6.9%) | 21920 (6.8%) |
| *35 to 44* | 32624 (10.3%) | 33008 (10.3%) |
| *45 to 54* | 48848 (15.5%) | 49005 (15.2%) |
| *55 to 64* | 63527 (20.1%) | 64619 (20.1%) |
| *65 to 74* | 74830 (23.7%) | 76656 (23.8%) |
| *75 to 84* | 44844 (14.2%) | 46836 (14.6%) |
| *85 or over* | 13373 (4.2%) | 13953 (4.3%) |
| *Missing* | 2607 (0.8%) | 2448 (0.8%) |
| **Gender** |  |  |
| *Male* | 124888 (39.5%) | 127069 (39.5%) |
| *Female* | 188370 (59.6%) | 191819 (59.6%) |
| *Missing* | 2784 (0.9%) | 2853 (0.9%) |
| **Ethnicity** |  |  |
| *White* | 269463 (85.3%) | 271904 (84.5%) |
| *Mixed* | 3168 (1.0%) | 3514 (1.1%) |
| *Asian* | 25712 (8.1%) | 27374 (8.5%) |
| *Black* | 8140 (2.6%) | 8690 (2.7%) |
| *Other* | 5158 (1.6%) | 5999 (1.9%) |
| *Missing* | 4401 (1.4%) | 4260 (1.3%) |
| **Working Status** |  |  |
| *Full-time paid work* | 85105 (26.9%) | 87851 (27.3%) |
| *Part-time paid work* | 40777 (12.9%) | 42237 (13.1%) |
| *Full-time education at school, college or university* | 6742 (2.1%) | 6640 (2.1%) |
| *Unemployed* | 9202 (2.9%) | 9309 (2.9%) |
| *Permanently sick or disabled* | 14910 (4.7%) | 15173 (4.7%) |
| *Fully retired from work* | 120045 (38.0%) | 122424 (38.1%) |
| *Looking after the home* | 16965 (5.4%) | 16841 (5.2%) |
| *Doing something else* | 8366 (2.6%) | 8523 (2.6%) |
| *Missing* | 13930 (4.4%) | 12743 (4.0%) |
| **Sexual Orientation** |  |  |
| *Heterosexual / straight* | 281066 (88.9%) | 286706 (89.1%) |
| *Gay / Lesbian* | 3516 (1.1%) | 4039 (1.3%) |
| *Bisexual* | 2174 (0.7%) | 2456 (0.8%) |
| *Other* | 2404 (0.8%) | 2643 (0.8%) |
| *I would prefer not to say* | 17442 (5.5%) | 17386 (5.4%) |
| *Missing* | 9440 (3.0%) | 8511 (2.6%) |
| **Religion** |  |  |
| *No religion* | 70300 (22.2%) | 75304 (23.4%) |
| *Buddhist* | 2013 (0.6%) | 2241 (0.7%) |
| *Christian* | 199504 (63.1%) | 197954 (61.5%) |
| *Hindu* | 6481 (2.1%) | 6885 (2.1%) |
| *Jewish* | 2153 (0.7%) | 2269 (0.7%) |
| *Muslim* | 14730 (4.7%) | 15941 (5.0%) |
| *Sikh* | 3251 (1.0%) | 3501 (1.1%) |
| *Other* | 3950 (1.2%) | 4064 (1.3%) |
| *I would prefer not to say* | 9850 (3.1%) | 10280 (3.2%) |
| *Missing* | 3810 (1.2%) | 3302 (1.0%) |
| **Indices of Deprivation** |  |  |
| *Most deprived* | 71650 (22.7%) | 72944 (22.7%) |
| *2* | 77035 (24.4%) | 78401 (24.4%) |
| *3* | 82020 (26.0%) | 82909 (25.8%) |
| *Least deprived* | 85337 (27.0%) | 87487 (27.2%) |
| **Rurality** |  |  |
| *Urban* | 249161 (78.8%) | 253825 (78.9%) |
| *Rural* | 66881 (21.2%) | 67916 (21.1%) |
| **Long-term health conditions** |  |  |
| *Yes* | 170911 (54.1%) | 175664 (54.6%) |
| *No* | 125174 (39.6%) | 125507 (39.0%) |
| *Don’t know/can’t say* | 8282 (2.6%) | 8666 (2.7%) |
| *I would prefer not to say* | 5253 (1.7%) | 5499 (1.7%) |
| *Missing* | 6422 (2.0%) | 6405 (2.0%) |
| **Total patients*** | 8553.13 (4915.9) | 8808.16 (5278.3) |
| **Total number of FTE GPs per 10,000 patients*** | 5.43441 (2.2) | 5.78009 (2.7) |
| **Total number of FTE Nurses per 10,000 patients*** | 2.64237 (1.9) | 2.65345 (1.8) |
| **Direct distance from home to their registered GP (km)*** | 1.97965 (2.3) | 1.99112 (2.6) |

**Standard deviation in parentheses*

**Appendix 5: Regression output in the main regression for days of a week service delivery model**

|  | **Overall experience to GP** | | **Satisfaction with appointment time** | | **Overall experience of making an appointment** | | **Frequencies**    **to see or speak to preferred GP** | |
| --- | --- | --- | --- | --- | --- | --- | --- | --- |
|  | **coef.** | **s.e.** | **coef.** | **s.e.** | **coef.** | **s.e.** | **coef.** | **s.e.** |
| **Number of extended access days per week** | -0.0000375 | (0.001) | 0.000402 | (0.001) | -0.000413 | (0.001) | -0.000617 | (0.001) |
| **Age** |  |  |  |  |  |  |  |  |
| *16-24 (base)* |  |  |  |  |  |  |  |  |
| *25 to 34* | 0.0138** | (0.005) | 0.0274*** | (0.005) | 0.0207*** | (0.005) | 0.0148** | (0.005) |
| *35 to 44* | 0.0391*** | (0.005) | 0.0403*** | (0.005) | 0.0350*** | (0.005) | 0.0134** | (0.005) |
| *45 to 54* | 0.0586*** | (0.005) | 0.0367*** | (0.005) | 0.0239*** | (0.005) | 0.0285*** | (0.005) |
| *55 to 64* | 0.0761*** | (0.005) | 0.0608*** | (0.005) | 0.0382*** | (0.005) | 0.0503*** | (0.005) |
| *65 to 74* | 0.0954*** | (0.005) | 0.112*** | (0.005) | 0.0788*** | (0.005) | 0.0741*** | (0.005) |
| *75 to 84* | 0.118*** | (0.005) | 0.171*** | (0.005) | 0.132*** | (0.005) | 0.0917*** | (0.006) |
| *85 or over* | 0.122*** | (0.005) | 0.190*** | (0.006) | 0.150*** | (0.006) | 0.0846*** | (0.007) |
| *Missing* | 0.0706*** | (0.013) | 0.0979*** | (0.013) | 0.0893*** | (0.014) | 0.0278* | (0.013) |
| **Gender** |  |  |  |  |  |  |  |  |
| *Malen(base)* |  |  |  |  |  |  |  |  |
| *Female* | -0.00518*** | (0.002) | -0.00669*** | (0.002) | -0.00929*** | (0.002) | -0.0317*** | (0.002) |
| *Missing* | -0.0131 | (0.011) | 0.00584 | (0.011) | -0.00623 | (0.012) | 0.00560 | (0.013) |
| **Ethnicity** |  |  |  |  |  |  |  |  |
| *White (base)* |  |  |  |  |  |  |  |  |
| *Mixed* | 0.00130 | (0.006) | 0.00190 | (0.008) | 0.00286 | (0.008) | -0.0243** | (0.009) |
| *Asian* | -0.0236*** | (0.005) | -0.0214*** | (0.006) | -0.0449*** | (0.006) | -0.0755*** | (0.005) |
| *Black* | 0.0452*** | (0.005) | 0.0692*** | (0.006) | 0.0452*** | (0.005) | -0.0813*** | (0.006) |
| *Other* | 0.00897 | (0.005) | 0.0597*** | (0.007) | 0.0276*** | (0.007) | -0.0561*** | (0.007) |
| *Missing* | -0.0281*** | (0.008) | -0.0328*** | (0.008) | -0.0470*** | (0.008) | -0.0258** | (0.009) |
| **Indices of Deprivation** |  |  |  |  |  |  |  |  |
| *Most deprived (base)* |  |  |  |  |  |  |  |  |
| *2* | 0.00230 | (0.003) | -0.00964** | (0.003) | -0.00363 | (0.003) | 0.00729* | (0.003) |
| *3* | 0.000811 | (0.003) | -0.0190*** | (0.003) | -0.00900** | (0.003) | 0.0125*** | (0.003) |
| Least deprived | 0.00490 | (0.003) | -0.0228*** | (0.004) | -0.00826* | (0.003) | 0.0154*** | (0.003) |
| **Working Status** |  |  |  |  |  |  |  |  |
| *Full-time paid work (base)* |  |  |  |  |  |  |  |  |
| *Part-time paid work* | 0.0264*** | (0.002) | 0.0578*** | (0.003) | 0.0369*** | (0.003) | 0.00347 | (0.002) |
| *Full-time education at school, college or university* | 0.0213*** | (0.006) | 0.0479*** | (0.007) | 0.0301*** | (0.007) | 0.0396*** | (0.007) |
| *Unemployed* | 0.0490*** | (0.004) | 0.128*** | (0.005) | 0.0875*** | (0.005) | 0.0492*** | (0.005) |
| *Permanently sick or disabled* | 0.0194*** | (0.003) | 0.0880*** | (0.005) | 0.0485*** | (0.004) | 0.0851*** | (0.004) |
| *Fully retired from work* | 0.0326*** | (0.002) | 0.0911*** | (0.003) | 0.0571*** | (0.003) | 0.0584*** | (0.003) |
| *Looking after the home* | 0.0235*** | (0.003) | 0.0807*** | (0.004) | 0.0510*** | (0.004) | 0.0222*** | (0.004) |
| *Doing something else* | 0.0165*** | (0.004) | 0.0637*** | (0.005) | 0.0377*** | (0.006) | 0.0410*** | (0.005) |
| *Missing* | 0.0228*** | (0.003) | 0.0815*** | (0.004) | 0.0491*** | (0.004) | 0.0300*** | (0.004) |
| **Sexual Orientation** |  |  |  |  |  |  |  |  |
| *Heterosexual / straight (base)* |  |  |  |  |  |  |  |  |
| *Gay / Lesbian* | -0.00879 | (0.006) | -0.0146* | (0.007) | -0.0171* | (0.007) | 0.0225** | (0.008) |
| *Bisexual* | 0.00544 | (0.007) | 0.0351*** | (0.010) | 0.0138 | (0.010) | 0.0323** | (0.011) |
| *Other* | 0.0312*** | (0.008) | 0.0806*** | (0.009) | 0.0601*** | (0.011) | -0.00641 | (0.012) |
| *I would prefer not to say* | -0.0000380 | (0.004) | 0.0383*** | (0.005) | 0.0165*** | (0.005) | -0.0300*** | (0.004) |
| *Missing* | 0.0225*** | (0.005) | 0.0672*** | (0.006) | 0.0408*** | (0.005) | -0.0374*** | (0.005) |
| **Religion** |  |  |  |  |  |  |  |  |
| *No religion (base)* |  |  |  |  |  |  |  |  |
| *Buddhist* | 0.0113 | (0.008) | 0.0459*** | (0.010) | 0.0207* | (0.010) | -0.00603 | (0.011) |
| *Christian* | 0.0226*** | (0.002) | 0.0433*** | (0.002) | 0.0323*** | (0.002) | -0.00588** | (0.002) |
| *Hindu* | 0.0299*** | (0.006) | 0.0368*** | (0.009) | 0.0424*** | (0.009) | 0.00632 | (0.007) |
| *Jewish* | 0.0108 | (0.009) | 0.0229 | (0.013) | -0.0103 | (0.015) | -0.00660 | (0.012) |
| *Muslim* | 0.00478 | (0.005) | 0.0202** | (0.006) | 0.00807 | (0.007) | -0.0115 | (0.006) |
| *Sikh* | 0.0160 | (0.009) | 0.0298** | (0.010) | 0.0267** | (0.010) | 0.0354*** | (0.010) |
| *Other* | -0.0165** | (0.006) | 0.00119 | (0.007) | -0.00701 | (0.007) | 0.00998 | (0.008) |
| *I would prefer not to say* | -0.0472*** | (0.005) | -0.0620*** | (0.005) | -0.0551*** | (0.006) | -0.0281*** | (0.006) |
| *Missing* | -0.0222** | (0.007) | -0.0279*** | (0.008) | -0.0349*** | (0.008) | -0.0188* | (0.009) |
| **Rurality** |  |  |  |  |  |  |  |  |
| *Urban (base)* |  |  |  |  |  |  |  |  |
| *Rural* | -0.00176 | (0.003) | -0.00851* | (0.004) | -0.00869* | (0.003) | 0.000221 | (0.004) |
| **Long-term health conditions** |  |  |  |  |  |  |  |  |
| *Yes (base)* |  |  |  |  |  |  |  |  |
| *No* | 0.0203*** | (0.001) | 0.0273*** | (0.002) | 0.0404*** | (0.002) | -0.0533*** | (0.002) |
| *Don’t know/can’t say* | -0.0420*** | (0.005) | -0.0545*** | (0.006) | -0.0371*** | (0.005) | -0.0752*** | (0.005) |
| *I would prefer not to say* | -0.0270*** | (0.007) | -0.0280*** | (0.007) | -0.0134 | (0.007) | -0.0339*** | (0.007) |
| *Missing* | 0.0204*** | (0.004) | 0.0458*** | (0.005) | 0.0472*** | (0.005) | -0.0284*** | (0.006) |
| **Year** |  |  |  |  |  |  |  |  |
| *Year 1 (base)* |  |  |  |  |  |  |  |  |
| *Year 2* | -0.00828*** | (0.002) | -0.0147*** | (0.002) | -0.0139*** | (0.002) | -0.0232*** | (0.002) |
| **Total patients** | -0.00000163* | (0.000) | -0.00000356*** | (0.000) | -0.00000543*** | (0.000) | -0.00000309** | (0.000) |
| **Total number of FTE GPs per 10,000 patients** | 0.000324 | (0.001) | 0.00176 | (0.001) | 0.00131 | (0.001) | 0.000535 | (0.001) |
| **Total number of FTE nurses per 10,000 patients** | 0.000189 | (0.002) | -0.00194 | (0.002) | -0.00182 | (0.002) | -0.00277 | (0.002) |
| **Direct distance from home to their registered GP (km)** | 0.00199*** | (0.000) | 0.00176*** | (0.000) | 0.00247*** | (0.000) | 0.00316*** | (0.001) |
| **Cons** | 0.784*** | (0.012) | 0.568*** | (0.017) | 0.665*** | (0.015) | 0.520*** | (0.015) |

*"* p<0.05 ** p<0.01 *** p<0.001"; N=*637783 in each regression. The effect of days of a week extended service delivery was estimated on each outcome measure, using linear probability models. Models controlled for two-way fixed effects, practices, and patients’ characteristics; standard errors are cluster at CCG level.

**Appendix 6: Regression output in the main regression for weekdays of a week service delivery model**

|  | **Overall experience to GP** | | **Satisfaction with appointment time** | | **Overall experience of making an appointment** | | **Frequencies**    **to see or speak to preferred GP** | |
| --- | --- | --- | --- | --- | --- | --- | --- | --- |
|  | **coef.** | **s.e.** | **coef.** | **s.e.** | **coef.** | **s.e.** | **coef.** | **s.e.** |
| **Number of extended access weekdays per week** | 0.0000492 | (0.001) | 0.000650 | (0.001) | -0.000570 | (0.001) | -0.000838 | (0.001) |
| **Age** |  |  |  |  |  |  |  |  |
| *16-24 (base)* |  |  |  |  |  |  |  |  |
| *25 to 34* | 0.0138** | (0.005) | 0.0274*** | (0.005) | 0.0207*** | (0.005) | 0.0148** | (0.005) |
| *35 to 44* | 0.0391*** | (0.005) | 0.0403*** | (0.005) | 0.0350*** | (0.005) | 0.0134** | (0.005) |
| *45 to 54* | 0.0586*** | (0.005) | 0.0367*** | (0.005) | 0.0239*** | (0.005) | 0.0285*** | (0.005) |
| *55 to 64* | 0.0761*** | (0.005) | 0.0608*** | (0.005) | 0.0382*** | (0.005) | 0.0503*** | (0.005) |
| *65 to 74* | 0.0954*** | (0.005) | 0.112*** | (0.005) | 0.0788*** | (0.005) | 0.0741*** | (0.005) |
| *75 to 84* | 0.118*** | (0.005) | 0.171*** | (0.005) | 0.132*** | (0.005) | 0.0917*** | (0.006) |
| *85 or over* | 0.122*** | (0.005) | 0.190*** | (0.006) | 0.150*** | (0.006) | 0.0846*** | (0.007) |
| *Missing* | 0.0706*** | (0.013) | 0.0979*** | (0.013) | 0.0893*** | (0.014) | 0.0278* | (0.013) |
| **Gender** |  |  |  |  |  |  |  |  |
| *Male (base)* |  |  |  |  |  |  |  |  |
| *Female* | -0.00518*** | (0.002) | -0.00669*** | (0.002) | -0.00929*** | (0.002) | -0.0317*** | (0.002) |
| *Missing* | -0.0131 | (0.011) | 0.00583 | (0.011) | -0.00623 | (0.012) | 0.00561 | (0.013) |
| **Ethnicity** |  |  |  |  |  |  |  |  |
| *White(base)* |  |  |  |  |  |  |  |  |
| *Mixed* | 0.00130 | (0.006) | 0.00190 | (0.008) | 0.00286 | (0.008) | -0.0243** | (0.009) |
| *Asian* | -0.0236*** | (0.005) | -0.0214*** | (0.006) | -0.0449*** | (0.006) | -0.0755*** | (0.005) |
| *Black* | 0.0452*** | (0.005) | 0.0692*** | (0.006) | 0.0452*** | (0.005) | -0.0813*** | (0.006) |
| *Other* | 0.00897 | (0.005) | 0.0597*** | (0.007) | 0.0276*** | (0.007) | -0.0561*** | (0.007) |
| *Missing* | -0.0281*** | (0.008) | -0.0328*** | (0.008) | -0.0470*** | (0.008) | -0.0258** | (0.009) |
| **Indices of Deprivation** |  |  |  |  |  |  |  |  |
| *Most deprived (base)* |  |  |  |  |  |  |  |  |
| *2* | 0.00230 | (0.003) | -0.00964** | (0.003) | -0.00363 | (0.003) | 0.00729* | (0.003) |
| *3* | 0.000812 | (0.003) | -0.0190*** | (0.003) | -0.00900** | (0.003) | 0.0125*** | (0.003) |
| Least deprived | 0.00490 | (0.003) | -0.0228*** | (0.004) | -0.00826* | (0.003) | 0.0154*** | (0.003) |
| **Working Status** |  |  |  |  |  |  |  |  |
| *Full-time paid work (base)* |  |  |  |  |  |  |  |  |
| *Part-time paid work* | 0.0264*** | (0.002) | 0.0578*** | (0.003) | 0.0369*** | (0.003) | 0.00348 | (0.002) |
| *Full-time education at school, college or university* | 0.0213*** | (0.006) | 0.0479*** | (0.007) | 0.0301*** | (0.007) | 0.0396*** | (0.007) |
| *Unemployed* | 0.0490*** | (0.004) | 0.128*** | (0.005) | 0.0875*** | (0.005) | 0.0492*** | (0.005) |
| *Permanently sick or disabled* | 0.0194*** | (0.003) | 0.0880*** | (0.005) | 0.0485*** | (0.004) | 0.0851*** | (0.004) |
| *Fully retired from work* | 0.0326*** | (0.002) | 0.0911*** | (0.003) | 0.0571*** | (0.003) | 0.0584*** | (0.003) |
| *Looking after the home* | 0.0235*** | (0.003) | 0.0807*** | (0.004) | 0.0510*** | (0.004) | 0.0222*** | (0.004) |
| *Doing something else* | 0.0165*** | (0.004) | 0.0637*** | (0.005) | 0.0377*** | (0.006) | 0.0410*** | (0.005) |
| *Missing* | 0.0228*** | (0.003) | 0.0815*** | (0.004) | 0.0491*** | (0.004) | 0.0300*** | (0.004) |
| **Sexual Orientation** |  |  |  |  |  |  |  |  |
| *Heterosexual / straight (base)* |  |  |  |  |  |  |  |  |
| *Gay / Lesbian* | -0.00879 | (0.006) | -0.0146* | (0.007) | -0.0171* | (0.007) | 0.0225** | (0.008) |
| *Bisexual* | 0.00545 | (0.007) | 0.0351*** | (0.010) | 0.0138 | (0.010) | 0.0323** | (0.011) |
| *Other* | 0.0312*** | (0.008) | 0.0807*** | (0.009) | 0.0601*** | (0.011) | -0.00641 | (0.012) |
| *I would prefer not to say* | -0.0000378 | (0.004) | 0.0383*** | (0.005) | 0.0165*** | (0.005) | -0.0300*** | (0.004) |
| *Missing* | 0.0225*** | (0.005) | 0.0672*** | (0.006) | 0.0408*** | (0.005) | -0.0374*** | (0.005) |
| **Religion** |  |  |  |  |  |  |  |  |
| *No religion (base)* |  |  |  |  |  |  |  |  |
| *Buddhist* | 0.0113 | (0.008) | 0.0459*** | (0.010) | 0.0207* | (0.010) | -0.00603 | (0.011) |
| *Christian* | 0.0226*** | (0.002) | 0.0433*** | (0.002) | 0.0323*** | (0.002) | -0.00588** | (0.002) |
| *Hindu* | 0.0299*** | (0.006) | 0.0368*** | (0.009) | 0.0424*** | (0.009) | 0.00631 | (0.007) |
| *Jewish* | 0.0108 | (0.009) | 0.0229 | (0.013) | -0.0103 | (0.015) | -0.00660 | (0.012) |
| *Muslim* | 0.00478 | (0.005) | 0.0202** | (0.006) | 0.00807 | (0.007) | -0.0116 | (0.006) |
| *Sikh* | 0.0160 | (0.009) | 0.0298** | (0.010) | 0.0267** | (0.010) | 0.0354*** | (0.010) |
| *Other* | -0.0165** | (0.006) | 0.00119 | (0.007) | -0.00701 | (0.007) | 0.00998 | (0.008) |
| *I would prefer not to say* | -0.0472*** | (0.005) | -0.0620*** | (0.005) | -0.0551*** | (0.006) | -0.0281*** | (0.006) |
| *Missing* | -0.0222** | (0.007) | -0.0279*** | (0.008) | -0.0349*** | (0.008) | -0.0188* | (0.009) |
| **Rurality** |  |  |  |  |  |  |  |  |
| *Urban (base)* |  |  |  |  |  |  |  |  |
| *Rural* | -0.00176 | (0.003) | -0.00851* | (0.004) | -0.00869* | (0.003) | 0.000221 | (0.004) |
| **Long-term health conditions** |  |  |  |  |  |  |  |  |
| *Yes (base)* |  |  |  |  |  |  |  |  |
| *No* | 0.0203*** | (0.001) | 0.0273*** | (0.002) | 0.0404*** | (0.002) | -0.0533*** | (0.002) |
| *Don’t know/can’t say* | -0.0420*** | (0.005) | -0.0545*** | (0.006) | -0.0371*** | (0.005) | -0.0752*** | (0.005) |
| *I would prefer not to say* | -0.0270*** | (0.007) | -0.0280*** | (0.007) | -0.0134 | (0.007) | -0.0339*** | (0.007) |
| *Missing* | 0.0204*** | (0.004) | 0.0458*** | (0.005) | 0.0472*** | (0.005) | -0.0284*** | (0.006) |
| **Year** |  |  |  |  |  |  |  |  |
| *Year 1 (base)* |  |  |  |  |  |  |  |  |
| *Year 2* | -0.00835*** | (0.002) | -0.0147*** | (0.002) | -0.0140*** | (0.002) | -0.0232*** | (0.002) |
| **Total patients** | -0.00000163* | (0.000) | -0.00000356*** | (0.000) | -0.00000543*** | (0.000) | -0.00000309** | (0.000) |
| **Total number of FTE GPs per 10,000 patients** | 0.000325 | (0.001) | 0.00176 | (0.001) | 0.00131 | (0.001) | 0.000538 | (0.001) |
| **Total number of FTE nurses per 10,000 patients** | 0.000184 | (0.002) | -0.00195 | (0.002) | -0.00182 | (0.002) | -0.00278 | (0.002) |
| **Direct distance from home to their registered GP (km)** | 0.00199*** | (0.000) | 0.00176*** | (0.000) | 0.00247*** | (0.000) | 0.00316*** | (0.001) |
| **Cons** | 0.783*** | (0.012) | 0.568*** | (0.017) | 0.666*** | (0.015) | 0.520*** | (0.015) |

*"* p<0.05 ** p<0.01 *** p<0.001"; N=*637783 in each regression. The effect of weekdays of a week extended service delivery was estimated on each outcome measure, using linear probability models. Models controlled for two-way fixed effects, practices, and patients’ characteristics; standard errors are cluster at CCG level.

**Appendix 7: Regression output in the main regression for weekends of a week service delivery model**

|  | **Overall experience to GP** | | **Satisfaction with appointment time** | | **Overall experience of making an appointment** | | **Frequencies**    **to see or speak to preferred GP** | |
| --- | --- | --- | --- | --- | --- | --- | --- | --- |
|  | **coef.** | **s.e.** | **coef.** | **s.e.** | **coef.** | **s.e.** | **coef.** | **s.e.** |
| **Number** of **extended access weekends per week** | -0.000588 | (0.002) | 0.0000803 | (0.002) | -0.000595 | (0.002) | -0.000966 | (0.002) |
| **Age** |  |  |  |  |  |  |  |  |
| *16-24 (base)* |  |  |  |  |  |  |  |  |
| *25 to 34* | 0.0138** | (0.005) | 0.0274*** | (0.005) | 0.0207*** | (0.005) | 0.0148** | (0.005) |
| *35 to 44* | 0.0391*** | (0.005) | 0.0403*** | (0.005) | 0.0350*** | (0.005) | 0.0134** | (0.005) |
| *45 to 54* | 0.0586*** | (0.005) | 0.0367*** | (0.005) | 0.0239*** | (0.005) | 0.0285*** | (0.005) |
| *55 to 64* | 0.0761*** | (0.005) | 0.0608*** | (0.005) | 0.0382*** | (0.005) | 0.0503*** | (0.005) |
| *65 to 74* | 0.0954*** | (0.005) | 0.112*** | (0.005) | 0.0788*** | (0.005) | 0.0741*** | (0.005) |
| *75 to 84* | 0.118*** | (0.005) | 0.171*** | (0.005) | 0.132*** | (0.005) | 0.0917*** | (0.006) |
| *85 or over* | 0.122*** | (0.005) | 0.190*** | (0.006) | 0.150*** | (0.006) | 0.0846*** | (0.007) |
| *Missing* | 0.0706*** | (0.013) | 0.0979*** | (0.013) | 0.0893*** | (0.014) | 0.0278* | (0.013) |
| **Gender** |  |  |  |  |  |  |  |  |
| *Male (base)* |  |  |  |  |  |  |  |  |
| *Female* | -0.00517*** | (0.002) | -0.00669*** | (0.002) | -0.00930*** | (0.002) | -0.0317*** | (0.002) |
| *Missing* | -0.0131 | (0.011) | 0.00586 | (0.011) | -0.00625 | (0.012) | 0.00557 | (0.013) |
| **Ethnicity** |  |  |  |  |  |  |  |  |
| *White (base)* |  |  |  |  |  |  |  |  |
| *Mixed* | 0.00130 | (0.006) | 0.00189 | (0.008) | 0.00286 | (0.008) | -0.0243** | (0.009) |
| *Asian* | -0.0236*** | (0.005) | -0.0214*** | (0.006) | -0.0449*** | (0.006) | -0.0755*** | (0.005) |
| *Black* | 0.0452*** | (0.005) | 0.0692*** | (0.006) | 0.0452*** | (0.005) | -0.0813*** | (0.006) |
| *Other* | 0.00897 | (0.005) | 0.0597*** | (0.007) | 0.0276*** | (0.007) | -0.0561*** | (0.007) |
| *Missing* | -0.0281*** | (0.008) | -0.0328*** | (0.008) | -0.0470*** | (0.008) | -0.0258** | (0.009) |
| **Indices of Deprivation** |  |  |  |  |  |  |  |  |
| *Most deprived (base)* |  |  |  |  |  |  |  |  |
| *2* | 0.00230 | (0.003) | -0.00964** | (0.003) | -0.00363 | (0.003) | 0.00729* | (0.003) |
| *3* | 0.000811 | (0.003) | -0.0190*** | (0.003) | -0.00900** | (0.003) | 0.0125*** | (0.003) |
| Least deprived | 0.00490 | (0.003) | -0.0228*** | (0.004) | -0.00826* | (0.003) | 0.0154*** | (0.003) |
| **Working Status** |  |  |  |  |  |  |  |  |
| *Full-time paid work (base)* |  |  |  |  |  |  |  |  |
| *Part-time paid work* | 0.0264*** | (0.002) | 0.0578*** | (0.003) | 0.0369*** | (0.003) | 0.00348 | (0.002) |
| *Full-time education at school, college or university* | 0.0213*** | (0.006) | 0.0479*** | (0.007) | 0.0301*** | (0.007) | 0.0396*** | (0.007) |
| *Unemployed* | 0.0490*** | (0.004) | 0.128*** | (0.005) | 0.0875*** | (0.005) | 0.0492*** | (0.005) |
| *Permanently sick or disabled* | 0.0194*** | (0.003) | 0.0880*** | (0.005) | 0.0485*** | (0.004) | 0.0851*** | (0.004) |
| *Fully retired from work* | 0.0326*** | (0.002) | 0.0911*** | (0.003) | 0.0571*** | (0.003) | 0.0584*** | (0.003) |
| *Looking after the home* | 0.0235*** | (0.003) | 0.0807*** | (0.004) | 0.0510*** | (0.004) | 0.0222*** | (0.004) |
| *Doing something else* | 0.0165*** | (0.004) | 0.0637*** | (0.005) | 0.0377*** | (0.006) | 0.0410*** | (0.005) |
| *Missing* | 0.0228*** | (0.003) | 0.0815*** | (0.004) | 0.0491*** | (0.004) | 0.0301*** | (0.004) |
| **Sexual Orientation** |  |  |  |  |  |  |  |  |
| *Heterosexual / straight (base)* |  |  |  |  |  |  |  |  |
| *Gay / Lesbian* | -0.00879 | (0.006) | -0.0146* | (0.007) | -0.0171* | (0.007) | 0.0225** | (0.008) |
| *Bisexual* | 0.00544 | (0.007) | 0.0351*** | (0.010) | 0.0138 | (0.010) | 0.0323** | (0.011) |
| *Other* | 0.0312*** | (0.008) | 0.0806*** | (0.009) | 0.0601*** | (0.011) | -0.00641 | (0.012) |
| *I would prefer not to say* | -0.0000370 | (0.004) | 0.0383*** | (0.005) | 0.0165*** | (0.005) | -0.0300*** | (0.004) |
| *Missing* | 0.0225*** | (0.005) | 0.0672*** | (0.006) | 0.0408*** | (0.005) | -0.0374*** | (0.005) |
| **Religion** |  |  |  |  |  |  |  |  |
| *No religion (base)* |  |  |  |  |  |  |  |  |
| *Buddhist* | 0.0113 | (0.008) | 0.0459*** | (0.010) | 0.0207* | (0.010) | -0.00604 | (0.011) |
| *Christian* | 0.0226*** | (0.002) | 0.0433*** | (0.002) | 0.0323*** | (0.002) | -0.00588** | (0.002) |
| *Hindu* | 0.0299*** | (0.006) | 0.0368*** | (0.009) | 0.0424*** | (0.009) | 0.00632 | (0.007) |
| *Jewish* | 0.0108 | (0.009) | 0.0229 | (0.013) | -0.0103 | (0.015) | -0.00659 | (0.012) |
| *Muslim* | 0.00478 | (0.005) | 0.0202** | (0.006) | 0.00807 | (0.007) | -0.0115 | (0.006) |
| *Sikh* | 0.0160 | (0.009) | 0.0298** | (0.010) | 0.0267** | (0.010) | 0.0354*** | (0.010) |
| *Other* | -0.0165** | (0.006) | 0.00120 | (0.007) | -0.00701 | (0.007) | 0.00998 | (0.008) |
| *I would prefer not to say* | -0.0472*** | (0.005) | -0.0620*** | (0.005) | -0.0551*** | (0.006) | -0.0281*** | (0.006) |
| *Missing* | -0.0222** | (0.007) | -0.0279*** | (0.008) | -0.0349*** | (0.008) | -0.0188* | (0.009) |
| **Rurality** |  |  |  |  |  |  |  |  |
| *Urban (base)* |  |  |  |  |  |  |  |  |
| *Rural* | -0.00176 | (0.003) | -0.00852* | (0.004) | -0.00869* | (0.003) | 0.000225 | (0.004) |
| **Long-term health conditions** |  |  |  |  |  |  |  |  |
| *Yes (base)* |  |  |  |  |  |  |  |  |
| *No* | 0.0203*** | (0.001) | 0.0273*** | (0.002) | 0.0404*** | (0.002) | -0.0533*** | (0.002) |
| *Don’t know/can’t say* | -0.0420*** | (0.005) | -0.0545*** | (0.006) | -0.0371*** | (0.005) | -0.0752*** | (0.005) |
| *I would prefer not to say* | -0.0269*** | (0.007) | -0.0280*** | (0.007) | -0.0133 | (0.007) | -0.0339*** | (0.007) |
| *Missing* | 0.0204*** | (0.004) | 0.0458*** | (0.005) | 0.0472*** | (0.005) | -0.0284*** | (0.006) |
| **Year** |  |  |  |  |  |  |  |  |
| *Year 1 (base)* |  |  |  |  |  |  |  |  |
| *Year 2* | -0.00815*** | (0.002) | -0.0144*** | (0.002) | -0.0141*** | (0.002) | -0.0235*** | (0.002) |
| **Total patients** | -0.00000163* | (0.000) | -0.00000356*** | (0.000) | -0.00000543*** | (0.000) | -0.00000308** | (0.000) |
| **Total number of FTE GPs per 10,000 patients** | 0.000321 | (0.001) | 0.00175 | (0.001) | 0.00131 | (0.001) | 0.000538 | (0.001) |
| **Total number of FTE nurses per 10,000 patients** | 0.000199 | (0.002) | -0.00192 | (0.002) | -0.00184 | (0.002) | -0.00280 | (0.002) |
| **Direct distance from home to their registered GP (km)** | 0.00199*** | (0.000) | 0.00176*** | (0.000) | 0.00247*** | (0.000) | 0.00316*** | (0.001) |
| **Cons** | 0.784*** | (0.012) | 0.570*** | (0.017) | 0.664*** | (0.015) | 0.519*** | (0.015) |

*"* p<0.05 ** p<0.01 *** p<0.001"; N=*637783 in each regression. The effect of weekends of a week extended service delivery was estimated on each outcome measure, using linear probability models. Models controlled for two-way fixed effects, practices, and patients’ characteristics; standard errors are cluster at CCG level.

**Appendix 8: Heterogeneous analyses: associations between the number of extended access days (by days, weekdays, and weekends of a week) and each outcome measure in a smaller sample without any missing data of all control variables.**

|  | **Overall experience to GP** | | **Satisfaction with appointment time** | | **Overall experience of making an appointment** | | **Frequencies** to **see or speak to preferred GP** | |
| --- | --- | --- | --- | --- | --- | --- | --- | --- |
|  | **coef.** | **s.e.** | **coef.** | **s.e.** | **coef.** | **s.e.** | **coef.** | **s.e.** |
| *All* | 0.0002 | (0.001) | 0.0000944 | (0.001) | -0.0006 | (0.001) | -0.0009 | (0.001) |
| *Weekdays* | 0.0004 | (0.001) | 0.000176 | (0.001) | -0.0009 | (0.001) | -0.0013 | (0.001) |
| *Weekends* | 0.0000 | (0.002) | -0.000105 | (0.003) | -0.0009 | (0.002) | -0.0011 | (0.002) |

*"* p<0.05 ** p<0.01 *** p<0.001"; N=565018* in each regression. The effect of different types of service delivery (days, weekdays, and weekends of a week) were estimated separately on each outcome measure, using linear probability models. Models controlled for two-way fixed effects, practices, and patients’ characteristics; standard errors are cluster at CCG level.

**Appendix 9: Additional analyses: associations between the number of extended access days in a week and each outcome measure by whether patients realized their GP’s extended opening time (on weekdays or on weekends)**

|  | **Overall experience to GP** | | **Satisfaction with appointment time** | | **Overall experience of making an appointment** | | **Frequencies**    **to see or speak to preferred GP** | |
| --- | --- | --- | --- | --- | --- | --- | --- | --- |
|  | **coef.** | **s.e.** | **coef.** | **s.e.** | **coef.** | **s.e.** | **coef.** | **s.e.** |
| Number of extended access weekdays | -0.0000437 | (0.001) | -0.000970 | (0.001) | -0.00160 | (0.001) | -0.00190 | (0.001) |
| Others (base) |  |  |  |  |  |  |  |  |
| Yes | 0.0451*** | (0.004) | 0.116*** | (0.006) | 0.0706*** | (0.006) | 0.0972*** | (0.007) |
| Interaction between answer (yes) and number of extended access weekdays | 0.000356 | (0.001) | 0.00149 | (0.002) | 0.00112 | (0.002) | -0.00124 | (0.002) |
| Number of extended access weekends | -0.00151 | (0.002) | -0.00457 | (0.003) | -0.00361 | (0.003) | -0.00364 | (0.002) |
| Others (base) |  |  |  |  |  |  |  |  |
| Yes | 0.0501*** | (0.007) | 0.114*** | (0.012) | 0.0777*** | (0.012) | 0.0987*** | (0.012) |
| Interaction between answer (yes) and number of extended access weekends | -0.00378 | (0.005) | 0.00282 | (0.008) | -0.00153 | (0.007) | -0.0117 | (0.007) |
|  |  |  |  |  |  |  |  |  |

*Whether patients realized their GPS’ extended opening time was captured from GPPS question “As far as you are aware, what general practice appointment times are available to you?” and extended access data about whether their registered practices or practice hub had extended access services. The category “Yes” in number of extended access weekdays was defined by patients who answered “after 6:30pm on a weekday” in GPPS question and their registered practice or hub provided extended access services. The category “Yes” in number of extended access weekends was defined by patients who answered “on a Saturday or on a Sunday” in GPPS question and their registered practice or hub provided extended access services.*

*"* p<0.05 ** p<0.01 *** p<0.001"; N=565018 in each regression. The effect of extended access service provision in whether patients realized their GPs’ extended access service was estimated by an interaction term between the number of extended access weekdays (or weekends) per week and whether patients realized their GPs’ extended access services, using linear probability models. Models controlled for the number of extended access days in a week, patients’ age groups, two-way fixed effects, practices, and patients’ characteristics), standard errors are cluster at CCG level.*

**Appendix 10: Additional analyses: associations between how extended access services were provided (through GP practices or GP groups) by the week, weekdays, and weekends and each patient experience**

|  | **Overall experience to GP** | | **Satisfaction with appointment time** | | **Overall experience of making an appointment** | | **Frequencies to speak to preferred GP** | |
| --- | --- | --- | --- | --- | --- | --- | --- | --- |
|  | **coef.** | **s.e.** | **coef.** | **s.e.** | **coef.** | **s.e.** | **coef.** | **s.e.** |
| **Extended access groups: Week (categories: "Practice only" as base category)** | | |  |  |  |  |  |  |
| *No extended* | 0.00164 | (0.005) | 0.00201 | (0.007) | 0.0114 | (0.006) | 0.0196** | (0.007) |
| *Practice only (base)* |  |  |  |  |  |  |  |  |
| *Group only* | 0.0135* | (0.005) | 0.0110 | (0.007) | 0.0122 | (0.007) | 0.00432 | (0.007) |
| *Group & practice overlap* | 0.00292 | (0.003) | 0.00562 | (0.004) | 0.000557 | (0.004) | 0.00356 | (0.004) |
| *Group & practice no overlap* | -0.00282 | (0.006) | -0.00519 | (0.008) | -0.00794 | (0.010) | -0.00981 | (0.007) |
| **Extended access groups: Weekdays (categories: "Practice only" as base category)** | | | |  |  |  |  |  |
| *No extended* | -0.000598 | (0.005) | -0.00585 | (0.007) | 0.00566 | (0.006) | 0.00696 | (0.006) |
| *Practice only (base)* |  |  |  |  |  |  |  |  |
| *Group only* | 0.00843 | (0.006) | 0.00218 | (0.007) | 0.00670 | (0.008) | 0.000238 | (0.007) |
| *Group & practice overlap* | 0.00235 | (0.003) | 0.00741 | (0.004) | 0.000690 | (0.004) | 0.00177 | (0.004) |
| *Group & practice no overlap* | 0.0185 | (0.012) | -0.00569 | (0.019) | -0.0186 | (0.012) | -0.0206 | (0.020) |
| **Extended access groups: Weekends (categories: "Practice only" as base category)** | | | |  |  |  |  |  |
| *No extended* | 0.0101 | (0.005) | 0.0112 | (0.008) | 0.00545 | (0.007) | 0.00761 | (0.007) |
| *Practice only (base)* |  |  |  |  |  |  |  |  |
| *Group only* | 0.00849 | (0.006) | 0.0141 | (0.008) | 0.00294 | (0.007) | 0.00368 | (0.007) |
| *Group & practice overlap* | 0.0113* | (0.004) | 0.0105 | (0.007) | 0.00436 | (0.007) | 0.00835 | (0.006) |
| *Group & practice no overlap* | 0.0283 | (0.023) | 0.0602** | (0.020) | 0.0534** | (0.019) | 0.0525 | (0.083) |

*A categorical variable with five categories was created to show the provision type during the week, weekdays, and weekends. The data was collected from extended access dataset. The five categories are no provision by either practices or group, provision only by practice, provision only by group, provision by both practice and group in the overlapped days, and provision by practice and group but the provision days are not overlapped.*

*"* p<0.05 ** p<0.01 *** p<0.001"; N=637783 in each regression. The effect of how extended access services were provided (through GP practices or GP groups) by the week, weekdays, and weekends on each outcome was estimated separately, using linear probability models. Models controlled for the number of extended access days in a week, patients’ age groups, two-way fixed effects, practices, and patients’ characteristics), standard errors are cluster at CCG level.*

**Appendix 11: Sensitivity Analyses: Associations between the non-parametric number of extended access days (by days, weekdays, and weekends of a week) and each outcome measure**

|  | **Overall experience to GP** | | **Satisfaction with appointment time** | | **Overall experience of making an appointment** | | **Frequencies**    **to see or speak to preferred GP** | |
| --- | --- | --- | --- | --- | --- | --- | --- | --- |
|  | **coef.** | **s.e.** | **coef.** | **s.e.** | **coef.** | **s.e.** | **coef.** | **s.e.** |
| **Number of extended access days (categories: 0 as base category)** | | |  |  |  |  |  |  |
| *1* | 0.00400 | (0.006) | -0.00113 | (0.007) | -0.00992 | (0.007) | -0.0195** | (0.007) |
| *2* | 0.0100 | (0.005) | 0.00739 | (0.008) | 0.00242 | (0.007) | -0.0157* | (0.007) |
| *3* | 0.00205 | (0.007) | 0.00365 | (0.008) | -0.00885 | (0.008) | -0.0172* | (0.008) |
| *4* | 0.00274 | (0.006) | 0.00788 | (0.009) | -0.000868 | (0.008) | -0.0157 | (0.009) |
| *5* | 0.000146 | (0.007) | -0.00574 | (0.009) | -0.0104 | (0.008) | -0.0250** | (0.008) |
| *6* | 0.00307 | (0.006) | 0.00233 | (0.009) | -0.0160* | (0.008) | -0.0211** | (0.008) |
| *7* | 0.00424 | (0.005) | 0.00577 | (0.007) | -0.00309 | (0.006) | -0.0142* | (0.006) |
| **Number of extended access weekdays (categories: 0 as base category)** | | | |  |  |  |  |  |
| *1* | 0.00942 | (0.005) | 0.00832 | (0.007) | -0.00245 | (0.007) | -0.00409 | (0.007) |
| *2* | 0.00319 | (0.006) | 0.00814 | (0.007) | 0.00232 | (0.007) | -0.00883 | (0.006) |
| *3* | 0.00478 | (0.006) | 0.00545 | (0.007) | -0.00255 | (0.007) | -0.00641 | (0.008) |
| *4* | 0.00548 | (0.006) | 0.00702 | (0.009) | -0.0103 | (0.007) | -0.0118 | (0.008) |
| *5* | 0.00361 | (0.004) | 0.00694 | (0.006) | -0.00232 | (0.005) | -0.00656 | (0.005) |
| **Number of extended access weekends (categories: 0 as base category)** | | | |  |  |  |  |  |
| *1* | -0.00572 | (0.004) | -0.00120 | (0.005) | -0.00517 | (0.006) | -0.00729 | (0.005) |
| *2* | -0.00164 | (0.003) | 0.0000458 | (0.005) | -0.00160 | (0.005) | -0.00250 | (0.004) |

*"* p<0.05 ** p<0.01 *** p<0.001"; N=*637783 in each regression. The effect of different types of service delivery (days, weekdays, and weekends of a week) were estimated separately on each outcome measure, using linear probability models. Models controlled for two-way fixed effects, practices, and patients’ characteristics; standard errors are cluster at CCG level.

**Appendix 12: Sensitivity Analyses: Associations between the number of extended access days per week and each outcome measure by a probit model**

|  | **Overall experience to GP** | | **Satisfaction with appointment time** | | **Overall experience of making an appointment** | | **Frequencies**    **to see or speak to preferred GP** | |
| --- | --- | --- | --- | --- | --- | --- | --- | --- |
|  | **coef.** | **s.e.** | **coef.** | **s.e.** | **coef.** | **s.e.** | **coef.** | **s.e.** |
| **Number of extended access days (continuous)** | | |  |  |  |  |  |  |
| Probit model | 0.0000148 | (0.001) | 0.000407 | (0.001) | -0.000352 | (0.001) | -0.000636 | (0.001) |
| N | 631198 | | 637704 | | 636970 | | 637748 | |

*"* p<0.05 ** p<0.01 *** p<0.001";* The effect of number of extended access days of a week on each outcome measure was estimated separately, using linear probability models. Models controlled for two-way fixed effects, practices, and patients’ characteristics; standard errors are cluster at CCG level.

**Appendix 13: Sensitivity Analyses: Associations between the number of extended access days per week and each outcome measure in unbalanced sample, sample with no GP registrations <1000 patient, and in a model with interactive fixed effect between time and CCGs**

|  | **Overall experience to GP** | | **Satisfaction with appointment time** | | **Overall experience of making an appointment** | | **Frequencies**    **to see or speak to preferred GP** | |
| --- | --- | --- | --- | --- | --- | --- | --- | --- |
|  | **coef.** | **s.e.** | **coef.** | **s.e.** | **coef.** | **s.e.** | **coef.** | **s.e.** |
| **Number of extended access days (continuous)** | | |  |  |  |  |  |  |
| Unbalanced sample (N=5678194) | -0.0000902 | (0.001) | 0.000331 | (0.001) | -0.000492 | (0.001) | -0.000558 | (0.001) |
| Interactive fixed effect (N=637783) | -0.0000971 | (0.001) | -0.000116 | (0.001) | -0.000840 | (0.001) | -0.00104 | (0.001) |
| Drop registered patients<1000 (N=637212) | -0.0000354 | (0.001) | 0.000404 | (0.001) | -0.000412 | (0.001) | -0.000617 | (0.001) |

"* p<0.05 ** p<0.01 *** p<0.001"; The effect of number of extended access days of a week on each outcome measure was estimated separately in an unbalanced sample, sample with no GP registrations <1000 patient, and in a model with interactive fixed effect between time and CCGs, using linear probability models. Models controlled for two-way fixed effects, practices, and patients’ characteristics; standard errors are cluster at CCG level.
